# Gametogenesis in *Plasmodium*: Delving Deeper to Connect the Dots

**DOI:** 10.3389/fcimb.2022.877907

**Published:** 2022-06-15

**Authors:** Manoswini Dash, Sherry Sachdeva, Abhisheka Bansal, Abhinav Sinha

**Affiliations:** ^1^ Parasite Host Biology, Indian Council of Medical Research (ICMR)-National Institute of Malaria Research, New Delhi, India; ^2^ Central Molecular Laboratory, Govind Ballabh (GB) Pant Institute of Postgraduate Medical Education and Research, New Delhi, India; ^3^ School of Life Sciences, Jawaharlal Nehru University, New Delhi, India

**Keywords:** malaria, *Plasmodium*, gametogenesis, xanthurenic acid, exflagellation, transmission

## Abstract

In the coming decades, eliminating malaria is the foremost goal of many tropical countries. Transmission control, along with an accurate and timely diagnosis of malaria, effective treatment and prevention are the different aspects that need to be met synchronously to accomplish the goal. The current review is focused on one of these aspects i.e., transmission control, by looking deeper into the event called gametogenesis. In the *Plasmodium* life cycle, gametocytes are the first life forms of the sexual phase. The transmission of the parasite and the disease is critically dependent on the number, viability and sex ratio of mature gametocytes and their further development inside mosquito vectors. Gametogenesis, the process of conversion of gametocytes into viable gametes, takes place inside the mosquito midgut, and is a tightly regulated event with fast and multiple rounds of DNA replication and diverse cellular changes going on within a short period. Interrupting the gametocyte-gamete transition is ought to restrict the successful transmission and progression of the disease and hence an area worth exploring for designing transmission-blocking strategies. This review summarizes an in-depth and up-to-date understanding of the biochemical and physiological mechanism of gametogenesis in *Plasmodium*, which could be targeted to control parasite and malaria transmission. This review also raises certain key questions regarding gametogenesis biology in *Plasmodium* and brings out gaps that still accompany in understanding the spectacular process of gametogenesis.

## Background

As per the current knowledge, malaria in humans can be caused by at least nine different species of *Plasmodium*, namely - *falciparum, vivax, malariae, ovale curtisi, ovale wallikeri*, (Sutherland et al., 2010)*, knowlesi, cynomolgi* (Ta et al., 2014)*, simian* (Deane, 1992; Brasil et al., 2017) and *brasilianum* (Lalremruata et al., 2015). However, the majority of the disease burden is attributed to *P. falciparum* (known for the majority of malaria-related deaths) and *P. vivax* (known to cause relapses); human infections from the latter four species are reported to be zoonotic (Ramasamy, 2014). The mortality and morbidity due to *P. falciparum* and *P. vivax* have significantly declined in recent times, owing to the improvements in diagnostic and treatment approaches and the implementation of strategies like ‘Global Vector Control Response’ (WHO, 2017) and ‘High Burden to High Impact initiatives’ by World Health Organization (WHO, 2019). According to the WHO’s latest World Malaria Report, there were an estimated 241 million cases and 0.6 million malaria deaths worldwide in 2020, representing about 14 million more cases and 69,000 more deaths in 2020 than that in 2019, (WHO, 2021). Approximately two-thirds of these additional deaths (47,000) were linked to disruptions of services during the COVID-19 pandemic (WHO, 2019; WHO, 2021). To add further, the number of cases and deaths has shown stagnancy in the last five years, which is a matter of concern.

Looking at the “*incidence to mortality ratio*” of malaria in the last two decades, it can be deciphered that currently available medications, which potentially target the asexual symptom-causing parasitic forms, effectively reducing mortality, are inadequate in preventing the disease transmission. Strategies to interrupt malaria transmission have played a key role in tackling the condition for all malaria-afflicted countries that have either successfully controlled malaria or are at the elimination phase. However, the existence of a transmissible reservoir of gametocytes in asymptomatic individuals can reintroduce malaria transmission in countries that have successfully controlled or even eliminated malaria (Schneider et al., 2006; Bousema et al., 2012; Lindblade et al., 2013; Lin et al., 2014).

Two epidemiological metrics have been defined for assessing malaria elimination. i) Basic reproductive number (R_0_): the anticipated number of vectors (malaria invertebrate hosts) infected by one generation of parasite from an infected vertebrate host (or vector) (Smith et al., 2007). An R_0_ of less than one is indicative of malaria elimination. The overall effectiveness of an elimination-targeted intervention is quantified by its ability to reduce R_0_, over one round of transmission (from host to vector to host), and is termed as the effect size (Lloyd-Smith et al., 2009; Griffin, 2015; Griffin, 2016). ii) Entomological inoculation rate (EIR) which is the average number of infective bites per person in a unit time and is defined as the product of the human biting rate (HBR; average number of times a human is bitten by mosquitoes in a unit time) and sporozoite infection rate (SIR; proportion of biting mosquitoes that have sporozoites in their salivary glands) (Macdonald G, 1957. The Epidemiology and Control of Malaria. London: Oxford University Press.; Blagborough et al., 2013). It has been stated that EIR has to be less than one bite per person to achieve malaria elimination (Ulrich et al., 2013). Parasite transmission can be either from mosquito-to-human (M→H) or human-to-mosquito (H→M) and hence interventions at these two interfaces that involve targeting either sporozoites (M→H) or gametocytes/gametes (H→M)) are likely to be effective in reducing R0 below 1 in addition to other intervention strategies.

## Transmission of *Plasmodium* (Human to a Mosquito)

*Plasmodium* biology is highly intricate, involving two hosts with strikingly dissimilar microenvironments, transformation into multiple life stages with a brief transition period, and evasion of the host defense mechanisms, to complete its life cycle. *Plasmodium* inhabits a range of vertebrates as its primary host where the asexual phase is carried out. At the same time, the dipterans of the genus *Anopheles* are the only invertebrate and definitive host where the sexual phase of the parasite takes place. Successful interchange of parasites between the primary and secondary hosts is essential to continue the life cycle. Therefore, the parasite stages that are exchanged between the hosts are crucial in the context of disrupting transmission.

Gametocytes and sporozoites are the *Plasmodium* stages, that are transferred from H→M and M→H, respectively and therefore, interventions targeting sporozoites (M→H) or gametocytes/gametes (H→M) stages of the parasite can effectively block transmission. Since sporozoites are the infective stage and are solely responsible for causing the infection in human, most studies have been done in the M→H interface, targeting sporozoites. While gametocytes do not appear to have any direct role in causing pathology to the vertebrate host and therefore relatively less explored, they play a major role in maintaining the malaria burden. Successful transmission of the parasite from vertebrate host to mosquito and progression to further stages depends upon many factors like (i) sex ratio and viability of gametocytes, (ii) host and vector defense mechanism, (iii) behavior and occupation of the host, which indirectly controls the exposure of the host to mosquito (iv) vector density, behavior and competence, and (v) HBR of mosquito. Therefore, the gametocytes inside the human host and the gametocytes and gametes inside the mosquitoes need to be removed for effective inhibition of transmission. However, attempts to restrict transmission by interrupting the growth and development of gametocytes and/or gametes are scanty due to a lack of knowledge about the detailed biology of transition from gametocytes to gametes, and downstream physiology inside the mosquito midgut. The other reasons could be the technical hurdles associated with inspection of the approaches interrupting gametogenesis. Therefore, the knowledge gap between the transition of the two parasite stages needs to be filled, both in terms of morphological and physiochemical aspects, for effective designing of transmission-blocking strategies targeting the H→M interface. Current explorations have improved our insights on gametogenesis. The present review will emphasize on what is known about gametogenesis, the knowledge gaps, and the possible and novel avenues for developing transmission-intervention strategies by interfering gametogenesis.

## Gametocytes: The Precursor Stage of Transmission at the Human-to-Mosquito Interface

**Figure 1:**
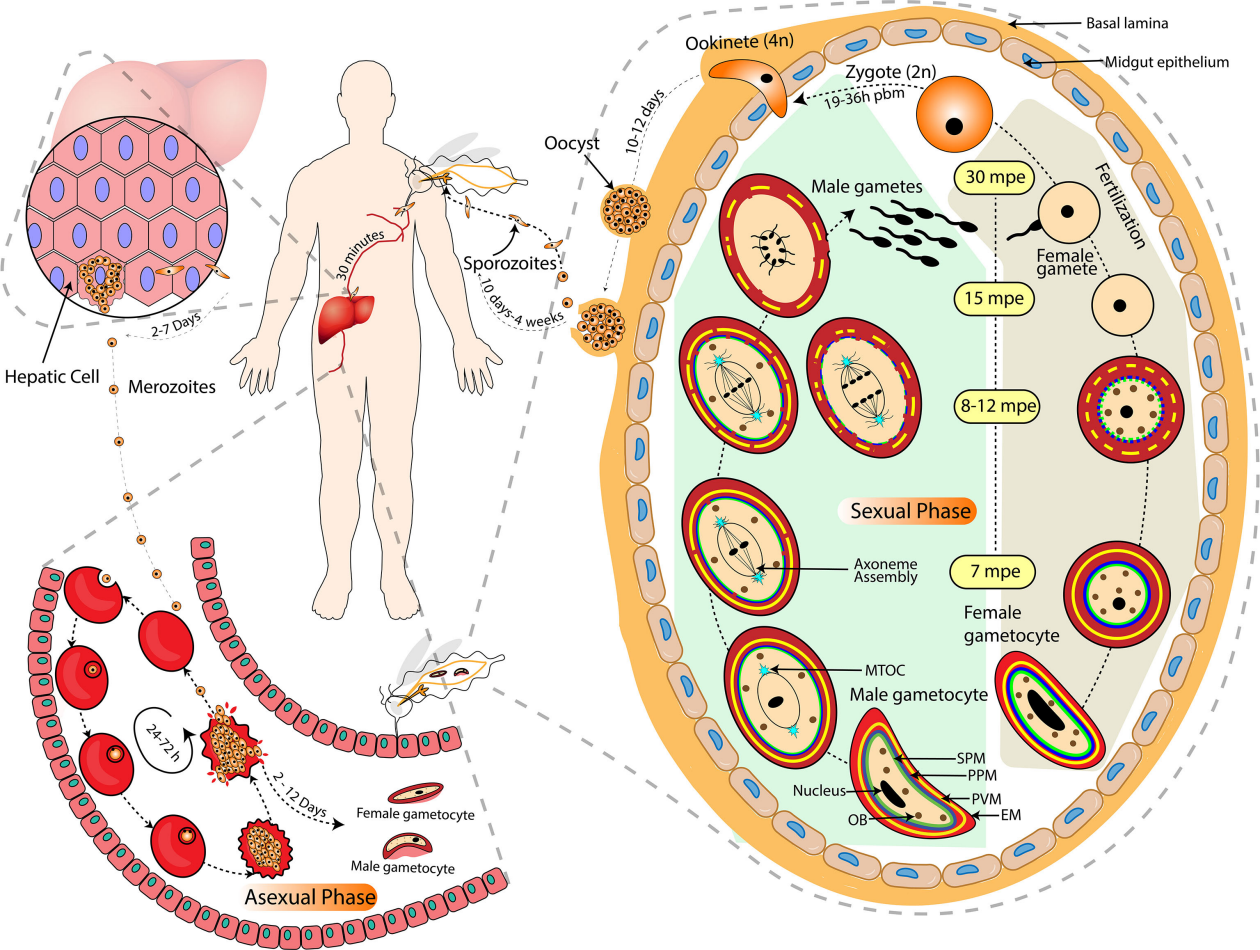
Life of Plasmodium in the Vertebrate and Invertebrate Host. The complete life cycle of *Plasmodium* is largely categorized into asexual (inside the mammalian host’s liver and RBCs) and sexual (including gametocytogenesis in the mammalian host’s RBCs and gametogenesis in the arthropod host’s midgut) phases. Activation of male gametocyte includes endomitosis, egress and exflagellation to release 8 motile microgametes whereas activation of female gametocytes includes maturation, rounding off and egress of single macrogamete. The approximate timing of each event after exposure to mosquito midgut environment is illustrated in terms of mpe (minutes post entry) and pbm (post blood meal). The grey dashed lines indicate a zoomed-in depiction of micro-events. The black arrow-headed dashed lines indicate the temporality and directionality of micro-events. EM, Erythrocyte membrane; PVM, Parasitophorus vacuole membrane; PPM, Parasite plasma membrane; SPM, Sub-pellicular membrane; MTOC, Microtubule Organizing Centre; OB, Osmiophilic Bodies.

Sexual stage commitment (or gametocytogenesis) is thought to occur in two ways. A subpopulation of merozoites is committed to developing into the sexual form i.e., gametocytes, without undergoing the erythrocytic schizogony inside the human host (Bancells et al., 2019). The other route involves gametocyte commitment at the erythrocytic schizont so that all the merozoites from a committed schizont are destined to develop into gametocytes of either sex but never both (Silvestrini et al., 2000; Poran et al., 2017) (**Figure 1**). Gametocyte commitment in *P. falciparum* is epigenetically regulated in which *P. falciparum* heterochromatin protein 1 (*Pf*HP1) is an essential factor for mitotic proliferation. Additionally, *Pf*HP1- dependent regulation of *Pf*AP2-G, a transcription factor initiated by de-repression of the ap2-g locus during DNA replication which regulates the switch from asexual proliferation to sexual differentiation (Brancucci et al., 2014; Kafsack et al., 2014; Waters, 2016; Poran et al., 2017; Llorà-Batlle et al., 2020). In a controlled human malaria infection (CHMI) by *P. falciparum*, less than 10% of the total asexual parasites are found to be committed to developing into gametocytes (Collins et al., 2018) while, in natural infection, less than 5% of the total parasite biomass are developed into mature gametocytes (Taylor and Read, 1997).

A committed merozoite upon sexual conversion by either pathway forms a gametocyte-committed ring or gametocyte ring, which is the first sexual developmental stage in the human host. Such rings then directly follow sexual development from stage I gametocyte until they reach maturity (stage V gametocytes) (Llorà-Batlle et al., 2020). The development of gametocytes in *P. falciparum* is completed in five stages, i.e., stage I-V, with distinct physiological and morphological changes (Hawking et al., 1971). The immature gametocytes are sequestered in host tissues, particularly in bone marrow and spleen, whilst only the mature gametocytes (stage V) are found in the peripheral blood circulation, ready to be taken up by the mosquitoes (Joice et al., 2014; De Niz et al., 2018; Lee et al., 2018; Obaldia et al., 2018). Protein synthesis and hemoglobin digestion cease in mature gametocytes approximately after six days of gametocyte development (Canning and Sinden, 1975; Sinden et al., 1978; Hayward, 2000; Baker, 2010) and gametocytes appear developmentally arrested at the G0 phase of the cell cycle (Canning and Sinden, 1975; Sinden et al., 1996). Nucleic acid synthesis is restricted to RNA synthesis, thereby disabling further genome replication in gametocytes (Raabe et al., 2009). Genome replication and cell division resumes only after activation of the gametocytes inside the mosquito midgut (Baker, 2010; Fang et al., 2017). Once ingested by a mosquito, each male gametocyte forms up to eight flagellated microgametes, whereas a female gametocyte develops into a single macrogamete in a process called gametogenesis. The physical and biochemical pathways involved in gametogenesis, as well as their importance and obstacles, will be the focus of this review.

## Fertilization, Zygote Formation and Ookinete Development

Fertilization of an immotile macrogamete by a microgamete involves the generation of slow flagellar waves and perpendicular orientation of the microgamete to the surface of macrogamete. The microgamete increases the frequency of the waves and continues to do so for around 14 secs to enter into the macrogamete (Sinden and Croll, 1975). Fertilization (fusion of the nuclei) is preceded by the fusion of the plasma membranes of male and female gametes; the axoneme and the adhered male nucleus enter into the cytoplasm of the macrogamete which leads to the formation of diploid zygote (fertilization) between 10 and 60 minutes post activation (mpa) (Janse et al., 1986). Male gametes contact the female gametes through *Pf*s48/45-P230 mediated cell-cell adhesion (van Dijk et al., 2001), and membrane fusion involves protein hapless 2 (HAP2) that ensures onward development of the parasite (Liu et al., 2008). The molecular mechanism of HAP2 that results in gamete fusion is still unknown. In *P. berghei*, it has been shown that a plant-like protein, Generative Cell-Specific 1 (*Pb*gcs1) gene, a highly conserved locus across all *Plasmodium* spp., is expressed only in microgametes and determines the male fertility and regulates the interaction of micro- and macrogametes, and hence makes an essential component of parasite fertilization (Hirai et al., 2008).

The nuclear fusion is followed by DNA replication and meiosis over the next three hours. It takes 19-36 hours post blood meal for parasites to convert into ookinete. The differentiation of zygote to ookinete stage involves meiosis and polarity development, producing tetraploid motile and polarized ookinete (Ranford-Cartwright et al., 1991).

The ookinete traverses through the mosquito’s midgut epithelial cell layer from the apical side to reach the basal lamina. Out of the five perforin like proteins present in *Plasmodium*, Perforin like Proteins 4 (PLP4) are female gametocyte-specific and have a role in ookinete development and traversal through the mosquito midgut. This invasion step is accompanied by host protective mechanisms that cause a considerable reduction in ookinete numbers (Han, 2000). However, little information is available on the considerable loss in the number of ookinetes, the surviving ookinetes transform into oocysts. The transition from ookinete to oocyst stage is one of the major bottlenecks of Plasmodium development. The oocyst grows extracellularly leading to formation of sporozoites, which are then released into the body cavity of the mosquito and enter its salivary glands (Hillyer et al., 2007). Sporozoites are transmitted during the mosquito bite into the mammalian host, wherein they initiate liver infection (**Figure 1**).

## The criticality of Gametogenesis and Its Biology

Gametogenesis is a compound process of cell differentiation and morphological changes likely engaging multiple pathways. Although the development of gametocytes circulating in the bloodstream of a vertebrate host is blocked in the G0 phase, they respond within seconds once they reach the mosquito midgut, which is also known as gametocyte activation. The male and female gametocytes then lyse and exit from the host erythrocytes. The female gametes are available for fertilization after emerging from the erythrocytes, while microgametes undergo various additional cellular actions at the same time to duplicate their genome three times and assemble their axoneme within 8-10 minutes of post-activation (Janse et al., 1986). Microgametes are finally expelled from the residual body of the male gametocyte in a spectacular process termed exflagellation (Laveran, 1880; Billker et al., 2004). Fertilization between male and female gametes occurs within 15 to 20 minutes of the generation of dimorphic gametes.

### Gametocyte Activation

The mature stage V gametocytes ingested by mosquitoes during their blood meal are exposed to an entirely different milieu that includes a decrease in temperature (from 37°C to around 20°C), increase in pH (from 7.4 to 8.0) and gametocyte activating factor (GAF), (Billker et al., 1997; Gregory E Garcia et al., 1998). Exposure to these changes triggers gametocyte activation, which is measured by the number of exflagellation centres. Of the three known environmental stimuli, decrease in temperature, along with one more factor (either pH or XA) is critically required for gametocyte activation (Sinden and Croll, 1975; Kawamoto et al., 1991). Therefore, pH and XA can be substituted with each other (Sinden et al., 1996; Billker et al., 1997). However, how these environmental signals, especially temperature and pH, are translated into biochemical stimuli is only partially understood, and the complete signaling cascade is yet to be deciphered.

#### Role of pH

The physiology of the mosquito midgut plays a vital role in gametogenesis. The pH (~7.4 to 8.0) experienced by the gametocyte inside the mosquito midgut is not its physiological pH, rather an increased one after a blood meal. The abdominal or posterior midgut of hematophagous insects usually maintains a pH of ~6 when not blood-fed (Santos et al., 2008). The alkaline medium of mosquito midgut after a blood meal is attributed to volatilization or hydration of CO_2_ released from blood in the presence of enzyme carbonic anhydrase (CA), which reduces the H^+^ concentration (thereby increasing pH) and produces HCO_3_^-^ ions (Nijhout and Carter, 1978; Billker et al., 2000). The HCO_3_^-^ ions generated during this process induce exflagellation *via* an ion-exchange mechanism (Kawamoto et al., 1992). Therefore, the rise of midgut pH subsequently increases the pH of the gametocyte or infected erythrocyte by activating the Na^+^/H^+^ exchange antiport system (Kawamoto et al., 1991). An increase in intracellular pH of the parasite leads to Ca^2+^ mobilization and cyclic guanosine monophosphate (cGMP) production (the cGMP mediated gametogenesis is explained in a later section), which explains how the change in pH regulates gametogenesis. The shift in pH does not have any role in DNA synthesis per se but affects other developmental events required for microgamete assembly and exflagellation (Kawamoto et al., 1991)

#### Role of Temperature

As mentioned earlier, numerous studies have shown that a drop in temperature is an inevitable factor for gametogenesis (Sinden and Croll, 1975; Sinden, 1983; Kawamoto et al., 1991; Ogwan’g et al., 1993; Billker et al., 1997), along with any one of the other factors, i.e., pH and/or GAF. The *Plasmodium* spp. complete their sporogonic development within the mosquito midgut in a temperature range of 16-24°C, with *P. berghei* at a minimum temperature of 16°C while *P. falciparum* at 18°C. The duration of the maturation period is thermodynamically controlled, i.e., the sporogony takes more time at lower temperatures, while at a higher temperature, the development process is relatively faster (Sinden and Croll, 1975). DNA synthesis during microgametogenesis occurs at 20° C and pH 7.3 but not at 37°C and 8.0 pH, suggesting that the enzymes involved in DNA synthesis might be thermodynamically regulated (Kawamoto et al., 1991). Even though the temperature is one of the indispensable changes the parasite encounters inside a mosquito, there are only studies in the late 90s showing how a rise in temperature affects the sporogonic development in mosquitoes and some speculations about how the temperature change regulates the process, but no solid shreds of evidence exist so far (Vanderberg and Yoeli, 1966; Kawamoto et al., 1991; Noden et al., 1995).

#### Role of Xanthurenic Acid

XA is a byproduct of the ommochrome pathway of tryptophan metabolism, which produces eye pigment in insects and is also known to play a major role in gametocyte activation (Summers et al., 1982; Garcia et al., 1998). XA is abundant inside the mosquito head, especially in the salivary gland (0.28+/-0.05 ng), and found in lower concentrations inside the midgut (0.05+/-0.01 ng) in a non-blood-fed mosquito. (Nijhout, 1979; Garcia et al., 1997; Okech et al., 2006). The reduction of XA concentration in the salivary gland immediately after a blood meal suggests that XA released in saliva during the blood meal might have entered into the midgut, therefore a lower concentration. of XA in the midgut than the salivary gland (Okech et al., 2006). However, further studies have shown a substantial increase in XA concentration immediately after a blood meal to millimolar scale (~ 5mM), which is thrice the magnitude of XA concentration in vertebrates (Lima et al., 2012). In blood-fed insects, XA functions as an antioxidant, reducing the oxidative stress generated by the heme or iron molecule after digestion of a blood meal. XA binds to both heme and iron and inhibits phospholipid oxidation, thereby acting as a protective molecule in mosquitoes (Lima et al., 2012; Yamamoto et al., 2018).

Although the role of an unknown mosquito factor in inducing gametogenesis was reported during the early nineties (Micks et al., 1948), its identity as a heat-stable and dialyzable molecule was first reported by Nijhout as “mosquito exflagellation factor (MEF)” (Nijhout, 1979). Later, the molecule was isolated from the gut lumen and head of the mosquito, characterized as a small and negatively charged chromophore and termed as “gametocyte activating factor (GAF)” (Garcia et al., 1997). In 1998, the compound was identified as XA (C_10_H_7_NO_4_), which can induce gametogenesis *in vitro* at a concentration lower than 0.5 µM in *P. berghei*, *P. gallinaceum* and *P. falciparum* (Billker et al., 1998; Garcia et al., 1998). An optimum infection is induced at a concentration of 100 µM in *P. falciparum* (Bhattacharyya and Kumar, 2001). The hydrophilic nature of XA suggests that it has ligand-like properties and interacts with receptors on gametocyte plasma membranes. However, the physiochemical process that stimulates gametogenesis is yet to be known. XA acts as a neurotransmitter in vertebrates and interacts with a specific G-protein coupled receptors (GPCR) to induce neuronal pathways (Taleb et al., 2012). A recent study in *P. yoelii* by exploiting all gametocyte integral membrane proteins revealed that a membrane protein called gametogenesis essential protein 1 (GEP1) interacts with guanylyl cyclase-α (GC-α) and induces cGMP- (protein kinase-G)PKG-Ca^2+^ signaling cascade (Jiang et al., 2020). The *Py*GEP1 is a transporter protein, expressed in cytoplasmic puncta of male and female gametocytes and conserved across the *Plasmodium* spp. Jiang et al. also reported similar cellular localization of GEP1 and GC-α in the cytoplasmic puncta. However, its direct role in sensing XA could not be established. Another G-protein coupled receptor protein found in *P. berghei* (*Pb*GPR180) is engaged in the cGMP-PKG-Ca^2+^ pathway and has a similar cellular location to GEP1 and GC- α (Wang et al., 2022). Although, there is a significant decrease in oocyst intensity and prevalence rate in the pbgpr180 deleted parasites however, sizable amount of infection in mosquitoes infected with pbgpr180 parasites suggest auxillary role of the gene in parasite development in mosquito. Though it is hypothesized that it senses the environment signal (Temperature, pH or XA) essential for gametocyte activation, the exact mechanistic contribution of the molecule is yet to be determined. As a result, the question of how XA stimulates gametogenesis remains unanswered.

The sensitivity of pH and XA towards amiloride, a Na^+^/H^+^ exchange system inhibitor (Kawamoto et al., 1991), suggests a similar or common mechanism is followed by both of the factors to induce physiological changes required for gametogenesis. It may also explain why, in addition to temperature, the presence of one of these components is sufficient for gametogenesis (Billker et al., 1997). The enzymes involved in DNA synthesis, erythrocyte membrane disruption and exflagellation might be temperature and pH-sensitive and thus get activated at alkaline pH and lower temperatures, present in the mosquito midgut (Kawamoto et al., 1991). However, the order and reciprocity of these environmental and chemical inputs to orchestrate gametogenesis have yet to be deciphered.

### Microgametogenesis

An enlarged nucleus characterizes mature microgametocytes with an electron-dense intra-nuclear body, the cytoplasm with very few endoplasmic reticula, mitochondria, apicoplast and osmiophilic bodies (Sinden, 1982). The kinetochores present inside the nucleus are coupled to the MTOC located on the cytoplasmic side of the nuclear envelope through a nuclear pore. A series of events (endomitosis, rounding up, exflagellation and egress) occurred in male gametocytes within a short period to produce eight motile microgametes. All these events are initiated more or less simultaneously in a single time frame; some of these events last less than a minute, while others last longer. As a result, it is technically challenging to demarcate the chronology of the events because most of them are initiated together and continue concurrently. Furthermore, the order and duration of events differ amongst *Plasmodium* species. We attempted to discuss each of the events under successive headings, as best we could, following their probable chronology.

#### Endomitosis

In *Plasmodium*, closed mitosis involves karyokinesis (nuclear division) without concomitant cytokinesis (Arnot et al., 2011; Gerald et al., 2011; Wall et al., 2018). In many organisms, the centrosome serves as MTOC during mitosis, with separate pairs of duplicated centrosomes forming the two poles of the mitotic spindle (Conduit et al., 2015). But *Plasmodium* has structurally discrete centrosomes that lack traditional centrioles. The spindle microtubules in *Plasmodium* originate from an MTOC known as centriolar plaque (CP), which resembles the spindle pole body (SPB) of Yeasts and *Dictyostelium* (Francia et al., 2015). The CP acts as the primary site for the formation of microtubules and determining their movement during mitosis (**Figure 1**).

The *Plasmodium* life cycle comprises two atypical mitotic processes: the first occurs during blood-stage schizogony and resembles endomitosis (Gerald et al., 2011), while the second occurs during the formation of microgametes in the mosquito midgut (Sinden, 1991). During microgametogenesis, male gametocyte undergoes three rounds of quick genome duplication, changing from haploid to octoploid genome, concurrent chromatin condensation and nuclear budding, with an intact nuclear envelope. In *P. falciparum*, the first nuclear division during microgametogenesis is complete within 7 minutes of post-activation (mpa), followed by rounding up and the second nuclear division within 8 to 12 mpa. The final nuclear division and exflagellation event coincide, releasing eight motile microgametes (Sinden et al., 1978; Sinden, 1991). The chromosome condensation and cytokinesis are mediated by an E3 ubiquitin ligase named anaphase-promoting complex 3 (APC3), through proteolysis of cell cycle regulators (Wall et al., 2018). Phosphoprotein phosphatase (PPP) plays a critical role in regulating mitosis in eukaryotes. Recently, an orthologue of eukaryotic PPP has been characterized in *P. falciparum* (PPP1) that interacts with kinesins and plays a significant role in spindle formation and is associated with kinetochores (Zeeshan et al., 2021). A highly co-regulated and coordinated expression of genes during the intra-erythrocytic developmental cycle governs DNA replication (Bozdech et al., 2003). During gametogenesis, mitosis is an extremely fast and well-controlled process. However, the molecular players involved in controlling continued DNA replication during exflagellation have not been completely delineated.

#### Exflagellation

Exflagellation is an exclusive phenomenon associated with male gametogenesis. This process takes place in the mosquito midgut where the activated male gametocytes undergo three rounds of rapid genome replication, to transition from haploid to octoploid. Each condensed haploid nucleus, along with its associated MTOC, basal body, axoneme, and flagellum, forms a microgamete that exits the main cellular body in a spectacular process known as exflagellation. (Sinden, 1991). The process starts with the violent commotion of the content of the gametocytes, along with endomitosis producing eight nuclei in the periphery of the cell (Garnham et al., 1967). Concurrently, the single MTOC present in the microgametocytes divides and transforms into eight basal bodies, one for each nucleus. These basal bodies act as nucleation centres from where axonemes are developed. The duration of exflagellation is determined by species, temperature, and other unknown factors. Although a single microgametocyte can produce up to eight microgametes, abnormal circumstances such as vigorous movement, the entanglement of the gametes, and inadequate attachment of the nucleus and flagellum, to name a few, can lower the number to four. (Sinden and Croll, 1975). In a recent study, Yahiya and the group developed a workflow to visualize the live microgametogenesis event in *P. falciparum* by live-cell fluorescence imaging, providing a clear insights into cytoskeletal rearrangement, DNA replication and segregation for exflagellation (Yahiya et al., 2022). According to a recent comparative transcriptome analysis study, the CCCH Zinc finger protein (ZNF4) was shown to have higher expression in mature male and female P. falciparum gametocytes, and its disruption resulted in downregulation of male gametocyte-enriched transcripts. It's an RNA-binding protein that helps with mRNA metabolism during gametogenesis, especially during exflagellation (Hanhsen et al. 2022).

#### Egress

Egress is an essential step in the life cycle of *Plasmodium*. *Plasmodium* parasites, alternating between intracellular and extracellular stages in which most parts of their life cycle parasites reside in the parasitophorous vacuole (PV) within the host cell. One of these stages is gametogenesis, in which the male and female gametocytes that were developed inside the PV in the host erythrocytes are egressed to form gametes that participate in fertilization. This egress process involves the rupturing of two membranes covering the parasites: i) the parasitophorous vacuole membrane (PVM), and ii) the erythrocyte membrane (EM) in which egress of gametocytes from host RBC and egress of gamete from gametocyte PVM. However, the sequence of membrane rupture is still unclear whether gametocytes egress first from the RBC or vice versa.

Two different models are proposed to explain the egress mechanism and directionality of rupture. In a very detailed study on *P. yoelii nigeriensis*, rupture of EM and rounding up of the gametocytes are presented as maturation of the gametocytes and happens within 7 minutes post-activation (mpa) followed by exflagellation (Sinden and Croll, 1975). While in *P. falciparum*, within a minute of activation, the PVM ruptures at multiple sites followed by disintegration of subpellicular membrane (SPM). At last, the EM ruptures at a single point, approximately 15 mpa, following the inside-out egress model (Sologub et al., 2011; Yahiya et al., 2022). Also, in this species the microgametocytes are aligned at the pore formed in the erythrocyte by a single spindle pole before egress (Yahiya et al., 2022). However, the factors that regulate the disruption of PVM at multiple sites and EM at a single site are still gaps to be filled. The inside-out egress model is the currently accepted mechanism to explain the egress of gametes from the infected RBCs. In *P. berghei*, a similar mode of egress has been reported, which starts with the swelling of the cell, rupture of PVM, discharge of content of the OBs, and finally rupture of EM *via* a single pore opening (Andreadaki et al., 2018). Accordingly, by the time EM ruptures, three round of DNA replication with flagellar development approaches completion and ultimately flagellated microgametes comes out by rupturing the erythrocyte membrane. Therefore, the usage of the term gametocyte egress creates ambiguity and hence it would be more appropriate to use “egress of gamete” rather than “egress of gametocyte”.

#### Molecular Mechanisms Involved in Egress

The egress in gametogenesis is controlled by proteins, localized in OBs. OBs are membrane-bound, electron-dense structures found in male and female gametocytes, with sex-specific distinctive features such as the female OBs are thrice compared to male OBs in size and density and mostly oval-shaped while male OBs are club-shaped. The OBs carry many secretory proteins essential for escape. They are clubbed beneath the plasma membrane releasing their content through exocytosis into the vacuolar space, thereby meditating the breakdown of PVM and EM (Ishino et al., 2020). The molecular mechanism behind egress of gamete has been studied by reverse-genetic approaches in *Plasmodium*, especially in *P. berghei*. The effector proteins essential for membrane rupture resides in OBs, and their release is dependent on intracellular Ca^2+^ level (Olivieri et al., 2015). Proteins associated with OB also show sex-specificity. G377 is expressed only in female gametocytes and regulates the development of OBs and has a role in egress. A G377 knockout strain in *P. falciparum* female gametocytes shows a reduced number of OBs with consequently reduced infectivity to mosquitoes (de Koning-Ward et al., 2008). However, a similar study in *P. berghei* supports the female specificity of G377 has also been implicated in delayed egress phenotype albeit dispensable for parasite (Olivieri et al., 2015). Proteins with specific roles in the egress of male gamete have also been characterized.

In *P. berghei*, an isoform of actin named actin II has a significant role in rupturing PVM of male gamete while no considerable role in egress of female gametes, confirming male specificity (Deligianni et al., 2011). One of the five perforin-like proteins (PPLP) of *P. berghei*, PPLP2supports the egress of gamete from the erythrocyte membrane (Deligianni et al., 2013). However, in the *pplp2* mutant parasites, the PVM ruptures commonly while the EM remains intact (Deligianni et al., 2013). While other OB-resident proteins, namely male development-1/protein of early gametocyte 3 (MDV1/PEG3) and gamete egress and sporozoite traversal (GEST), are reported to express in both male and female gametes with more abundance in female gametes (Lal et al., 2009; Ponzi et al., 2009; Talman et al., 2011; Olivieri et al., 2015). Recently, thrombospondin related adhesive protein (TRAP)-like protein () called merozoite TRAP-like protein (MTRAP), residing in OBs has a significant role in egress of both sexes, and in its absence, gametes were trapped inside the host cell (Kehrer et al., 2016). Another study established the role of MTRAP in both *P. berghei* and *P. falciparum* gametogenesis by showing that in mutants parasites with disrupted *mtrap* neither the PVM nor the EM was ruptured in both the sexes (Talman et al., 2011; Wirth and Pradel, 2012). Similarly, GEP in *P. berghei* has been identified and shown to play an important role in egress of both the sexes and exflagellation in male gametes (Andreadaki et al., 2020). Lack of GEP causes aberrant rupture of both EM and PVM and delayed discharge of OBs (Andreadaki et al., 2020). The co-localization of the mentioned proteins in OBs and partially in other cellular parts, suggests a significant role of OBs in sexual stage egress.

### Macrogametogenesis

The mature female gametocyte (in comparison to the male gametocyte) has a smaller nucleus, higher number of osmophilic bodies and an extensive endoplasmic reticulum (ER) with high-density ribosomes required for active protein synthesis and subsequent development (Sinden, 1982; Khan et al., 2005). The maturation of the female gametocyte thus is a preparatory phase for the widespread protein synthesis with female gametocyte activation. Furthermore, in the female gametocyte, there is a large pool of female-specific mRNA, which is bound by a conserved translation repression protein complex consisting of development of zygote inhibited (DOZI) and CAR-I/Trailer Hitch Homolog (CITH) (Mair, 2006; Mair et al., 2010). These translationally repressed mRNA in storage will not be translated, hence post-activation, female gametocytes do not undergo genome replication, and give rise to a single, large round-shaped and motionless haploid macrogamete. Recently, it is reported that transcription factors of Apicomplexan Apetala 2 family (ApiAP2) member AP2-O3, also specifically active in the female gametocytes, is a transcription repressor that regulates the formation of female gamete (Li et al., 2021). In addition, recently, *Plasmodium yoelii* negative on TATA-less1-G (*Py*NOT1-G) was discovered to play dual and opposed sexual functions - reducing gametocyte commitment but supporting male (exflagellation) and female (preparing the female gamete for further development) gametogenesis (Hart et al., 2021). Though *Py*NOT1-G is a key part of mRNA de-adenylation complex (Ccr4p-associated factor/carbon catabolite repression4/negative on TATA-less (CAF1/CCR4/NOT)) and plays a fundamental role in preserving the mRNAs that are critical to *Plasmodium* sexual and early mosquito stage development, the mRNAs is regulated by the complex during gametogenesis is yet to be found (Hart et al., 2021).

### Molecular Mechanisms in Gametogenesis

**Figure 2:**
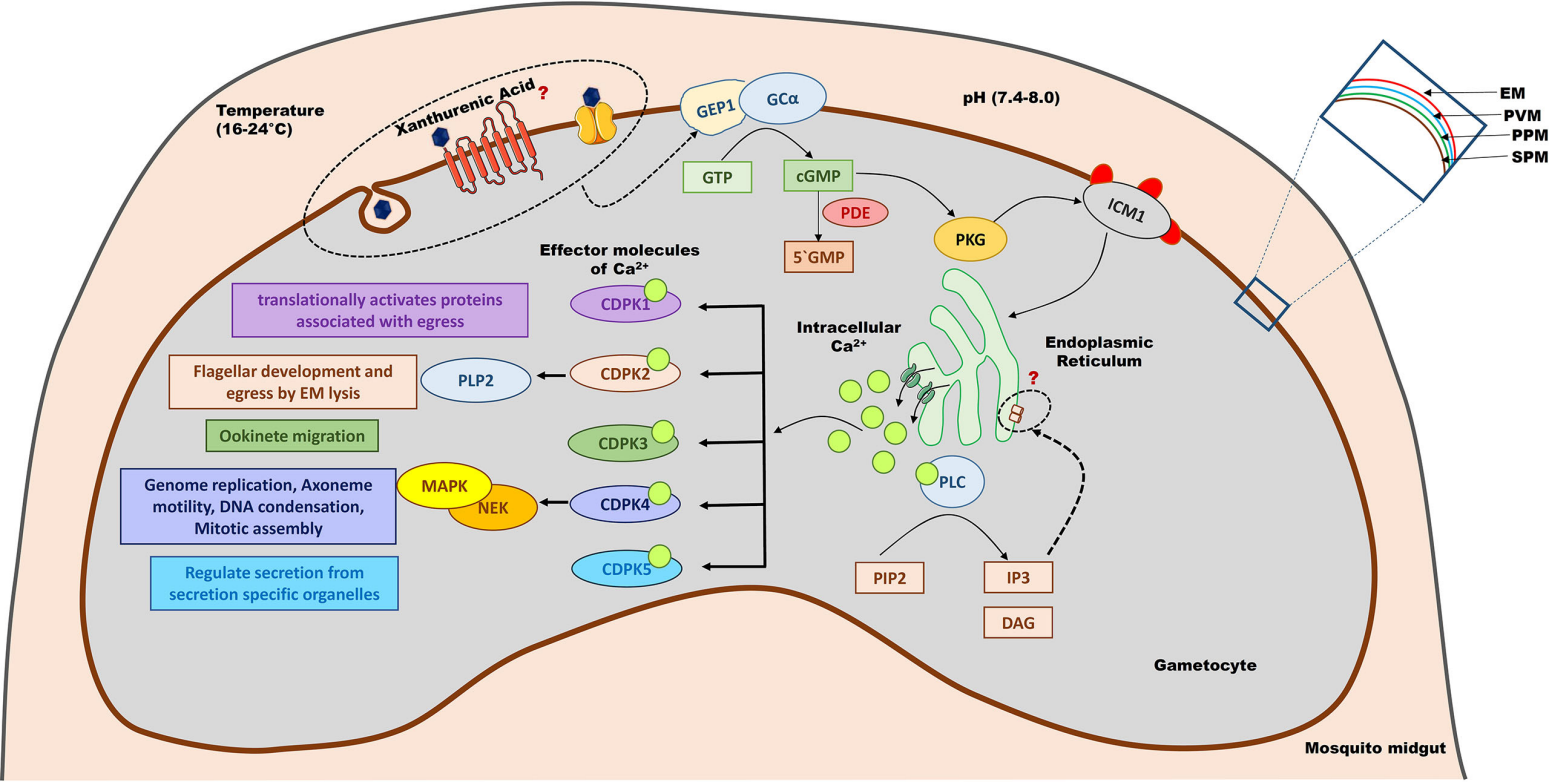
Signalling pathways involved in gametogenesis. Gametocytes are activated when exposed to an altered environment (lower temperature, higher pH and xanthurenic acid) of the mosquito midgut. Xanthurenic acid interacts with an unknown receptor or channel, bringing a structural change in GEP1 and thereby activating GCα. GCα converts GTP to cGMP, which in turn activates PKG. PKG phosphorylates ICM1 that would activate calcium channels present on the cellular organelles like mitochondria to release Ca^2+^ ions. Ca^2+^ also activates PLC, which catalyzes PIP2 to IP3 and DAG. IP3 further regulates calcium channels to maintain the intracellular calcium levels. Ca^2+^ acts as a secondary messenger to activate different CDPKs that modulate gametogenesis. CDPK5 regulates the secretion of essential protein molecules stored in secretory organelles like microneme and OBs to orchestrate gametogenesis. CDPK4 control the cell division event, and CDPK2 helps in flagellar development and EM lysis, during male gametogenesis. CDPK1 plays a major role in the egress of gametes. CDPK3 has a role in the later stage of gametogenesis and helps in ookinete migration to traverse the midgut cells to reach the basal lamina. GEP1, gametogenesis essential protein 1; GCα, Guanylyl Cyclase alpha; GTP, Guanosine triphosphate; cGMP, cyclic guanosine monophosphate; PDE, phosphodiesterase; PKG, Protein Kinase G; ICM1, Important for Ca2^+^ mobilization 1; PLC, Phospholipase C; PIP2, phosphatidylinositol (4,5)-bi-phosphate; IP3, Inositol-(1,4,5)-trisphosphate; DAG, Diacylglycerol; CDPK, calcium-dependent protein kinases; PLP2, Perforin-like proteins 2; MAPK, mitogen-activated protein kinases; NEK, NIMA like kinases; EM, Erythrocyte membrane; PVM, Parasitophorus vacuole membrane; PPM, Parasite plasma membrane; SPM, Sub-pellicular membrane.

The effect of temperature and pH in gametogenesis were mostly explored during the twentieth century. Later, multiple methodologies were used to examine the involvement of XA in triggering a signalling cascade for gametogenesis and sporogony development. Three effector pathways have been shown to orchestrate gametogenesis, either independently or in coordination *via* secondary messengers (**Figure 2**). The following sections go over the biochemical pathway(s) that XA uses to regulate gametogenesis.

#### cGMP as a Secondary Messenger

The crucial role of cGMP as a secondary messenger and the associated signaling pathway during gametogenesis has been evaluated by exploiting GC and phosphodiesterase (PDE) activity (Kawamoto et al., 1990; Kawamoto et al., 1991; Muhia et al., 2001; McRobert et al., 2008). The intracellular cGMP level in eukaryotes is regulated by GC and PDE by synthesizing and hydrolyzing cGMP, respectively. In *P. falciparum*, there are two membrane-associated GC (*Pf*GCα and *Pf*GCβ) (Carucci et al., 2000) and four putative cyclic nucleotide PDEs (PDE α-δ), of which *Pf*GCα and PDE- δ have significant roles during gametogenesis (Taylor et al., 2008; Baker et al., 2017). Enhanced activity of membrane-associated GCα in the presence of XA at 100 μM concentration was reported, by measuring the level of cGMP production (Muhia et al., 2001). However, whether XA stimulates GCα by directly interacting with it or *via* any other intermediate molecule(s) is yet to be deciphered. GEP1, a novel protein reported to work upstream of cGMP by inducing a conformational change in GCα, at the very initial stages of gametocyte activation in *P. yoelii*. *Py*GEP1 is exclusive to gametocytes and shown to be a critical for gametogenesis as GEP1 deficient strains could not developed exflagellation centres (EC) (Jiang et al., 2020). Other GPCR molecules are recently been identified to work upstream of cGMP in *P. berghei* and conserved across *Plasmodium* (Wang et al., 2022). Inhibition of PDE activity using pharmacological agents stimulates rounding up of mature gametocytes in the absence of XA (McRobert et al., 2008). Deletion of the gene coding for PDE-δ leads to an impaired ability for gametogenesis, due to premature increase in cGMP level; hence, the tight regulation of intracellular-cGMP level is critical for gametocyte activation (Taylor et al., 2008). These studies strongly indicate that regulation of intracellular cGMP level *via* GCs and PDEs is crucial for rounding up and exflagellation events (Taylor et al., 2008).

An increase of cGMP triggers the activation of cGMP-dependent PKG that controls the synthesis of phosphatidylinositol (4,5)-bi-phosphate (PIP_2_) (Brochet et al., 2014). Inhibition of PKG has been shown to dysregulate phosphoinositide metabolism leading to defects in egress of the parasite from red blood cells and ookinete motility (Brochet et al., 2014). Phosphatidylinositol specific phospholipase C (PI-PLC) is one of the classical effector molecules of intracellular Ca^2+^ signaling that hydrolyze PIP_2_ into diacylglycerol (DAG) and inositol-(1,4,5)-trisphosphate (IP_3_) (Berridge et al., 2000). While DAG activates protein kinase C (PKC), IP_3_ plays an important role in Ca^2+^ mobilization. It regulates the efflux of Ca^2+^ from cellular stores like ER and other organelles into the cytoplasm (Martin et al., 1994; Raabe et al., 2011), which acts as intracellular messenger and bind with effector molecules to amplify the signal and promotes gametogenesis (McRobert et al., 2008). Thus, activation of PKG leads to an increase in intracellular Ca^2+^ levels (Brochet et al., 2014). Cross-talk and interdependency between secondary messengers is a complex phenomenon in eukaryotes. Here, cGMP and Ca^2+^ act together to regulate the phosphoinositide metabolism *via* PKG and PI-PLC effector molecules to control gametogenesis. XA-mediated activation of gametocytes results in rounding up of both male and female gametocytes with a concomitant increase in cytosolic calcium level (McRobert et al., 2008) that perhaps leads to the activation of calcium-dependent protein kinases (discussed in the subsequent section). A quantitative global phosphoproteomics analysis of *P. falciparum* suggests cGMP/PKG as a signaling hub, that phosphorylates the proteins involved in different cellular events associated with nuclear division, egress and cell motility (Alam et al., 2015).

#### Role of Ca^2+^ Signaling

Ca^2+^ is the ubiquitous cellular secondary messenger in various organisms and regulates diverse signaling events. The intracellular Ca^2+^ signals are translated into cellular responses *via* a family of protein kinases named calcium-dependent protein kinases (CDPKs) that regulate different events of gametogenesis (Sebastian et al., 2012). The efflux mechanism to maintain cellular calcium levels is controlled either by Ca^2+^ channels or *via* receptors. In eukaryotes, there are various channels: two-pore channels (TPRs) and transient receptor potential (TRP) channels and receptors: inositol 1,4,5-triphosphate receptor (IP3R) and Ryanodine receptors (RyR), that helps in maintaining the intracellular Ca^2+^ level. However, in *Plasmodium*, a similar model of Ca^2+^ signaling is not validated yet. For example, even though cGMP-PKG and PI-PLC’s role in initiating and elevating Ca^2+^ mobilization during gametogenesis are well documented, the IP_3_ receptor (IP3R) is not yet identified in *Plasmodium* (Garcia et al., 2017). Few TPR channel homologs were successfully located while searching for the mammalian orthologs of Ca2+ channels and receptors in *Plasmodium*. However, receptors of IP_3_ and Ryanodine could not be identified yet (Prole and Taylor, 2011), which suggests that either the receptor is yet to be characterized or *Plasmodium* might have specific exclusive machinery to govern Ca^2+^ mobilization. In general, Apicomplexans lack many important calcium channels and related proteins, that further supports the existence of a distinctive calcium influx-reflux mechanism in *Plasmodium*, fewer Ca^2+^ channel-related genes are present compared to other eukaryotes as well as other Apicomplexan like *T. gondii. P. falciparum* is known to have more than 60 calcium-binding proteins (CBP), characterized by the presence of conserved structural helix-loop-helix motif, known as EF-hand domain. However, most of them are hypothetical (Budu et al., 2016). In a recent study, a multipass membrane protein named important for Ca^2+^ mobilization 1 (ICM1) was phosphorylated by PKG and regulated calcium mobilization from cellular stores in both asexual and sexual stages of the parasite (Balestra et al., 2021). However, whether ICM1 is the receptor of IP_3_ or part of a *Plasmodium*-specific calcium channel is still to be confirmed.

#### Role of CDPKs

A cascade of protein kinases activated in the Ca^2+^ signaling pathways have a crucial role at many stages of the *Plasmodium* life cycle, including the sexual stages (Ghartey-Kwansah et al., 2020). The Apicomplexan CDPKs have three functional domains namely, Ser/Thr kinase domain (KD), junction domain (JD) and CDPK activation domain (CAD). When Ca^2+^ binds to CDPK, the kinase refolds, exposing the KD to interact with the substrate. In *P. berghei*, 14 protein kinases are characterized to be involved in sexual development. In *P. falciparum*, the CDPK family has 7 members (CDPK 1-7), of which CDPK1 plays a vital role in gametocyte egress and zygote development by translationally activating the messenger RNAs (mRNAs), which are translationally repressed in macrogametes by mRNA ribonucleoproteins (mRNP) like the RNA helicase DOZI and the Sm-like factor CITH (Sebastian et al., 2012). CDPK1 in *P. falciparum* is shown to play a critical role in gametocyte egress from red blood cells. CDPK1 depleted parasites show a defect in egress of both the male gametes and female gametes. Moreover, CDPK1 KO female gametocytes do not round up post-induction (Bansal et al., 2018). CDPK2 is known to have a significant role in the development of flagella and RBC membrane lysis (Bansal et al., 2017). Interestingly, while CDPK1 seems essential for both male and female gametogenesis, CDPK2 appears to be specifically involved in the male gametogenesis phenomenon (Bansal et al., 2017). CDPK3 has a role in the later stages of sexual development, i.e. during migration of ookinete through mosquito midgut, however dispensable in gametogenesis (Bennink et al., 2016). In *P. berghei*, CDPK4 controls three distinct events during male gametogenesis, within 30 secs to1 min of activation. The parasites devoid of CDPK4 fails to undergo DNA synthesis, a prerequisite for the subsequent three mitotic divisions that leads to the formation of eight male gametes (Billker et al., 2004). It helps in the first round of genome replication, mitotic spindle assembly, DNA condensation, cytokinesis and axoneme motility (Fang et al., 2017). The role of CDPK4 in *P. falciparum* (*Pf*CDPK4) in exflagellation was studied using a chemical genetic approach, (Ojo et al., 2012; Ojo et al., 2014). A recent study unraveled the significant role of *Pf*CDPK4 in DNA replication, mRNA translation and cell motility (Kumar et al., 2021). The study also identified the putative substrates by identifying hypophosphorylated protein residues in *Pfcdpk4^-^* strain compared to wild-type. The list also includes GEP1, which is recently characterized to interact with XA (Jiang et al., 2020). However, *Pf*CDPK4 has no significant role in female gametogenesis. CDPK5 in *P.falciparum* (*Pf*CDPK5) plays an important role in the egress of parasites by regulating the release of molecules from secretion-specific organelles like OBs and microneme during the erythrocytic and pre-erythrocytic stages (Absalon et al., 2018; Govindasamy and Bhanot, 2020). However, there is no direct evidence for CDPK5 playing a role in egress of gametes from activated gametocytes.

The downstream activation of CDPK inducing mitogen-activated protein kinases (MAPK) regulate the events of DNA replication, axoneme assembly, nuclear division and egress of the gametes (Sinden, 2009). In a recent study, *P. falciparum* specific MAP kinase 2 (*Pf*MAP-2) is deciphered as an essential component for genome condensation, axoneme beating and cytokinesis, but does not have any role in genome replication. The study further revealed that *Pf*MAP-2 regulates these events by phosphorylating the target(s) involved in gametogenesis but not by controlling gene expression (Hitz et al., 2020). *Pf*MAP-2 is an atypical MAPK, with Thr-Ser-His activation site, which is different from the activation site usually found in proteins of the MAPK family. The *Pf*MAP-2 is known to be phosphorylated by NIMA (never in mitosis/Aspergillus) like kinases (*Pf*Nek-1) (Dorin et al., 2001). The enzymes belong to the Nek family and have a critical role in regulating mitosis and meiosis, localized in the spindle pole body (SPB), the MTOC in *Plasmodium*. In *P. falciparum*, four protein kinases of the Nek family (*Pf*Nek1-4) have been characterized, of which *Pf*Nek-1 is expressed only in male gametocytes (Dorin-Semblat et al., 2011), while *Pf*Nek-2 and *Pf*Nek-4 are expressed in both male and female gametocytes (Reininger et al., 2005; Reininger et al., 2009). The synergistic role of *Pf*Nek-2 and *Pf*MAP-2 to activate exogenous substrates is suggested but not scrutinized further.

Subsequently, the loss of two proteins – i) MAP-2 and ii) cell division cycle protein 20 (CDC20), hinders chromatin condensation, axoneme motility and cytokinesis, thereby inhibiting the formation of the male flagellar microgametes (Rangarajan et al., 2005; Tewari et al., 2005; Guttery et al., 2012). Also, exhaustion of the Metallo-dependent protein phosphatase PPM1 in *P. berghei* causes impaired exflagellation (Guttery et al., 2014), while reducing the Ca^2+^-dependent phosphatase calcineurin affects male gametogenesis and subsequent fertilization in the rodent malaria parasite (Philip and Waters, 2015). Recently, another protein in *P. berghei*, *Pb*22 is characterized to have an essential function in male gametogenesis by producing defective male gametes, which further affects egress, fertilization and ookinete formation (Liu et al., 2021). Though *Pb*22 is expressed in the membrane of both male and female gametes, it has no significant role in female gametogenesis, as proved by the cross-fertilization of male and female gametes of the *Pb*22KO line with wild strains (Liu et al., 2021).

## Gaps and Challenges

It would be off beam to state that the research concerning sexual stages of *Plasmodium* is scarce. There are plenty of studies that effectively identified many essential and stage-specific proteins that play a significant role in gametogenesis in different *Plasmodium* spp. Unfortunately, we still lack a clearer picture of the complete pathway. Needless to say, one of the factors driving these challenges include the complex life cycle of the parasite involving completely different life forms and stages. Peculiar to this is gametogenesis, which is comparatively more complex than any other *Plasmodium* life cycle stages as it involves many concurrent physiochemical events that are driven by multiple signaling cascades and happening in two sexually dimorphic cells. The order and synchronization of the signaling pathways associated with gametogenesis are partially solved. With the help of different molecular techniques like reverse genetics, chemical genetics, phosphoproteome and transcriptome analysis, more and more genes and proteins are being identified to play essential roles in *Plasmodium* gametogenesis. However, at the same time, it is becoming difficult to fit them at their respective places in the complex signaling pathways involved in gametogenesis. Reverse genetic studies are still one of the most powerful techniques to identify the genes and proteins critical for gametogenesis, the problem is that they are unable to decode the mechanisms of action of these identified critical molecules. Hence, despite significant development in the identification of genes, proteins and other molecules involved in gametogenesis, they seem to resemble numerous dots of a dot-puzzle that seldom do not contextually connect and pose difficulties in deciphering the ‘bigger picture’ needed to solve the gametogenesis puzzle. Many dynamically intriguing questions that preclude the connection between dots still prevail, including: (i) Whether reduced temperature, increase in pH and XA act independently, together, or in tandem? If independently, which of them is the most potent trigger? If in tandem, which of these is a precursor event? (ii) Are these triggers sex-specific? (iii) How does the reduced temperature and/or increase in pH translate into chemical stimuli to initiate gametogenesis? (iv) Although GEP has been identified to interact with XA to regulate activation very recently, it is yet to be found out whether GEP1 binds to GCα directly or some other intermediate molecules facilitate the pathway; (v) How do the secondary messengers interact with each other to orchestrate the complete signaling cascade? (vi) What is/are the specific receptor/s for IP3 that is/are critical regulator/s of intracellular calcium release?

## Concluding Remarks

There have been significant declines, at least in some countries, in malaria-related morbidity and mortality over the last few decades, that have shifted the focus of current research from attenuation of disease towards developing new transmission-blocking strategies to reduce transmission and achieve elimination of malaria. This will not be possible without having effective vaccine/s and drugs to prevent malaria transmission. For this to effectively happen, the parasites’ sexual stages in particular, the gametocyte-gamete transition, (also called gametogenesis) represent a key bottleneck and an ideal niche for identifying novel targets and translating them into effective public health strategies to interrupt transmission.

Although recent developments in dissecting and deciphering gametogenesis, including the complex phenomenon of gametocyte activation and its signaling pathways, have led to the identification of novel targets, there still exist critical knowledge gaps that preclude a more complete understanding of gametogenesis and its intricacies. Some of these gaps have been highlighted here but it can be unarguably concluded that there is an urgent need to delve deeper into this niche and connect the dots to be able to timely develop and deploy effective molecules into the drive for malaria elimination and eradication.

## Author Contributions

MD and SS contributed equally to the preparation of the MS. AS conceptualized the work, MD and SS acquired and interpreted the data, drafted, edited and reviewed the manuscript, MD generated the final version of figures, AB and AS critically reviewed and edited the MS for critical intellectual inputs. All authors contributed to designing the workflow, approved the final MS for publication, and agreed to be accountable for all aspects of the work.

## Funding

This work is supported by the Indian Council of Medical Research, New Delhi, India. MD was supported by Senior Research Fellowship (Grant ID: 45-2014/Geno-BMS) from the Indian Council of Medical Research, New Delhi, India and SS were supported by National Post-doctoral Fellowship (Grant ID: PDF/2017/001325) from the Department of Science and Technology- Science and Engineering Research Board (DST-SERB), New Delhi, India.

## Conflict of Interest

The authors declare that the research was conducted in the absence of any commercial or financial relationships that could be construed as a potential conflict of interest.

## Publisher’s Note

All claims expressed in this article are solely those of the authors and do not necessarily represent those of their affiliated organizations, or those of the publisher, the editors and the reviewers. Any product that may be evaluated in this article, or claim that may be made by its manufacturer, is not guaranteed or endorsed by the publisher.
